# *Sicyos angulatus* ameliorates acute liver injury by inhibiting oxidative stress via upregulation of anti-oxidant enzymes

**DOI:** 10.1080/13510002.2018.1546986

**Published:** 2018-11-14

**Authors:** Hyun-Yong Kim, Jung-Ran Noh, Sung-Je Moon, Dong-Hee Choi, Yong-Hoon Kim, Kyoung-Shim Kim, Hong-Sun Yook, Jin-Pyo An, Won-Keon Oh, Jung Hwan Hwang, Chul-Ho Lee

**Affiliations:** a Laboratory Animal Resource Center, Korea Research Institute of Bioscience and Biotechnology (KRIBB), Daejeon, Republic of Korea; b University of Science and Technology, Daejeon, Republic of Korea; c Department of Food and Nutrition, Chungnam National University, Daejeon, Republic of Korea; d Research Institute of Pharmaceutical Sciences, College of Pharmacy, Korea Bioactive Natural Material Bank, Seoul National University, Seoul, Republic of Korea

**Keywords:** Antioxidants, apoptosis, reactive oxygen species, liver, oxidative stress, injury, enzyme, extracts

## Abstract

**Objective:** We aimed to investigate the effect of *Sicyos angulatus* (SA) ethanolic extracts as antioxidants and potential treatments for liver disease.

**Methods:** To establish a mouse model of liver injury, C57BL/6 male mice were injected via the caudal vein with a single dose of concanavalin A (Con A, 15 mg kg^−1^). SA extracts were administered once by oral gavage 30 min before Con A injection.

**Results:***In vitro* studies showed that SA decreased *tert*-butyl hydroperoxide (*t*-BHP)-induced reactive oxygen species (ROS) production. SA administration reduced plasma alanine aminotransferase (ALT) and aspartate aminotransferase (AST) levels, as well as hepatic ROS levels, in a dose-dependent manner. Moreover, SA increased the activities of the hepatic antioxidant enzymes superoxide dismutase, catalase, and glutathione peroxidase in a dose-dependent manner. Furthermore, SA treatment reduced pro-apoptotic protein levels. Con A-mediated cytosolic release of Smac/DIABLO and apoptosis-inducing factor (AIF), which are markers of necrosis, were dramatically decreased in HepG2 cells treated with SA.

**Conclusion:** SA ameliorated liver injury and might be a good strategy for the treatment of liver injury.

## Introduction

The liver is a major organ that is exposed to bacterial products, toxins, and food-derived antigens [1]. Its failure causes critical consequences for metabolite detoxification, protein synthesis, metabolism, and digestive biochemical production [1]. There are many diseases that affect the liver, including alcoholic, fatty, and drug-induced liver diseases, as well as viral hepatitis [2].

Reactive oxygen species (ROS) oxidize cellular proteins, lipids, and nucleic acids, which leads to general cellular damage and dysfunction and may initiate cell death through various signaling cascades [3]. ROS generation is a major factor in the pathogenesis of many liver diseases [4,5]. Hepatocytic protein, lipid, and DNA are primarily affected by ROS, resulting in functional abnormalities in the liver [6]. The first line of defense against environmental challenges and injury is the innate immune system, which is activated much more rapidly than the adaptive immune system [2]. Its excessive activation is known to induce liver damage by increasing the production of cytokines, such as tumor necrosis factor-alpha (TNF-α) [7]. Liver injury induced by TNF-α has been proposed to involve the generation of ROS derived from either mitochondrial or non-mitochondrial sources [8]. Therefore, antioxidant therapy alone or in combination with other strategies appears to be a potential treatment for various liver diseases.

*Sicyos angulatus* (SA) is a problematic, invasive vine and its aggressive attacks on summer crops are well-known [9]. We found that the ethanolic extracts of SA exert protective effects against atherosclerosis by inhibiting proinflammatory cytokine production in rodents [10]. In this study, we investigated the antioxidant effect of SA extract and its use as a treatment for liver injury.

## Materials and methods

### Animals

Male 8-week-old C57BL/6J mice were maintained at the Korea Research Institute of Bioscience and Biotechnology (Daejeon, Korea). The mice were housed in a temperature-controlled room (22 ± 1°C) and exposed to 12 h light/dark cycle with unlimited food and water. The mice were randomly divided into four groups: (1) Normal group; (2) Con A group; (3) Con A + SA100 group; (4) Con A + SA300 group; (5) SA300 group. SA extracts were administered once by oral gavage 30 min before Con A (Sigma-Aldrich, St. Louis, MO, USA) injection. Mice were injected via the caudal vein with a single dose of 15 mg/kg Con A. Six hours after Con A injection, the mice were euthanized by cervical dislocation. The livers were excised, frozen in liquid nitrogen, and stored at –80°C. All animal experiments were approved by the Institutional Animal Care and Use Committee (IACUC, KRIBB-AEC-16095) and were performed in accordance with the institutional guidelines at KRIBB.

### Preparation of the SA extract

The SA extract was prepared and supplied by the Korea Bioactive Natural Material Bank (Seoul, Korea). Briefly, the dried aerial parts of SA were extracted three times with 70% ethanol for 6 h at room temperature. Afterward, the 70% ethanol-soluble extract was filtered and exhaustively concentrated under reduced pressure to produce a 70% ethanolic extract. The yield of the SA extract was 11%. This SA extract was subsequently suspended in 0.5% carboxymethyl cellulose (CMC) to a final concentration of 50 mg/ml as a stock solution. The working solution of SA was adjusted to the desired concentrations for use in the *in vitro* and *in vivo* experiments.

### ROS measurements

HepG2 cells were cultured in Dulbecco’s modified Eagle’s medium (DMEM, Hyclone, Logan, UT, USA) containing 10% fetal bovine serum supplemented with 100 U/ml penicillin and 100 μg/ml streptomycin, and they were incubated at 37°C in an atmosphere with 5% CO_2_. HepG2 cells were pretreated with DMSO 100 μg/ml or SA 300 μg/ml for 2 h. Next, medium in each well was replaced with fresh medium containing 0.3 mM *tert*-butyl hydroperoxide (*t*-BHP, Sigma-Aldrich) and 50 μM 2,7-dichlorofluorescein diacetate (DCF-DA, Invitrogen, Eugene, OR, USA), and the cells were incubated for 30 min. Fluorescence images were visualized using a fluorescence microscope (Nikon, Eclipse TE 2000-U, Japan). Fluorescence intensity was measured using a Victor3 1420 Multilabel Counter (Perkin-Elmer, Palo Alto, CA, USA) at an excitation of 485 nm and an emission of 530 nm, and the readings were normalized to the protein content of the corresponding wells.

For liver extract preparation, liver tissue was fractionated via homogenization with a tight-fitting pestle in 0.25 mol/l sucrose buffer. The homogenates were centrifuged at 600 × *g* for 10 min to remove the nuclear fraction, and the remaining supernatant was centrifuged at 10,000 × *g* for 20 min to obtain the mitochondria pellet. The remaining supernatant was centrifuged at 100,000 × *g* for 1 h to obtain the cytosolic supernatant for ROS measurements.

To determine total ROS, liver tissue extracts (100 μl) were incubated with 20 μM DCF-DA and the fluorescence was recorded. To determine H_2_O_2_ level, liver tissue extracts (100 μl) were incubated with 20 μM amplex red (Sigma-Aldrich) and 0.1 U/ml horseradish peroxidase (HRP; Sigma-Aldrich) and the fluorescence was recorded at 530 nm (excitation) and 620 nm (emission). Finally, to determine $O_{2}^{-}$ level, liver tissue extracts (100 μl) were incubated with 20 μM dihydroethidium (DHE; Sigma-Aldrich) and the fluorescence was recorded at 485 nm (excitation) and 620 nm (emission). All reactions were incubated at 37°C for 60 min. Fluorescence intensity was recorded using a Victor3 Multilabel Counter and was normalized to that of protein content.

### Plasma analysis

Blood samples were collected from the retro-orbital venous sinuses of the mice. Plasma was obtained by centrifugation of the blood at 10,000 × *g* for 5 min at 4°C. Alanine aminotransferase (ALT) and aspartate aminotransferase (AST) levels were measured using an automatic chemical analyzer (AU480, Beckman Coulter, Brea, USA).

### Antioxidant enzyme assay

The mitochondrial fraction is used for catalase (CAT) and superoxide dismutase (SOD) assays, and the cytosolic fraction is for glutathione peroxidase (GPx) and glutathione reductase (GR) assays. CAT activity was measured using Aebi’s method [11]. GPx activity was assayed using the Paglia and Valentine method [12]. GR activity was determined using the method of Pinto and Bartley [13], in which NADPH oxidation was monitored at 340 nm. SOD activity was measured using a superoxide dismutase (SOD) activity assay kit (Biovision, Milpitas, CA, USA).

### Histopathology

The livers were removed from the mice and immediately fixed in a buffer solution containing 10% formalin for pathologic analysis. Fixed tissues were processed for paraffin embedding, and 5-μm sections were prepared and stained with hematoxylin and eosin (H&E). For the analysis of cell death, apoptotic cells were detected using a TUNEL staining kit (ApopTag® Peroxidase *In Situ* Apoptosis Detection Kit, Millipore, Billerica, USA) according to the manufacturer’s protocol. For neutrophil staining, the sections were incubated with the primary antiserum (Santa Cruz, CA, USA) against neutrophils. Next, anti-rat IgG was used as the secondary antibody (Vectastain Elite HRP ABC kit, Vector Laboratories, Burlingame, CA, USA). Neutrophils were visualized by 3,3-diaminobenzidine (Peroxidase substrate kit, Vector Laboratories) staining.

### Western blotting

HepG2 cells were lysed by adding SDS sample buffer [62.5 mM Tris-HCl (pH 6.8), 6% (w/v) SDS, 30% glycerol, 125 mM dithiothreitol (DTT), and 0.03% bromophenol blue]. Cell lysates were separated using 10% SDS-polyacrylamide gel electrophoresis (PAGE) and were transferred to polyvinylidene difluoride (PVDF) membranes. The membranes were then incubated with primary antibodies against Bax (Bcl-2-associated X protein, Cell Signaling Technology, Danvers, MA, USA), Bcl-2 (B-cell lymphoma 2, Cell Signaling Technology), Bad (Bcl-2-associated death promoter, Cell Signaling Technology), and Bim (Bcl-2-like protein 11, Cell Signaling Technology). The secondary antibodies were HRP-conjugated goat anti–rabbit IgG and rabbit anti–mouse IgG.

For fractionation, HepG2 cells were homogenized in lysis buffer [210 mM mannitol, 70 mM sucrose, 1 mM EGTA, and 5 mM Hepes (pH 7.2)]. Lysates were centrifuged at 600 × *g* for 10 min at 4°C. The resulting supernatants were centrifuged at 17,000 × *g* for 10 min at 4°C to isolate cytosolic proteins. Total protein was quantified by the Bradford assay. Cytosolic fractions were used for western blotting using primary antibodies. Smac/DIABLO (second mitochondria-derived activator of caspase/direct inhibitor of apoptosis binding protein with low pI, Abcam, Cambridge, MA, USA), cytochrome C (Cell Signaling Technology), AIF (apoptosis-inducing factor, Cell Signaling Technology), and β-actin were used as a loading control.

### Statistical analysis

All data are presented as the mean ± standard error of the mean (SEM). Significant differences between 2 groups were determined using Student’s *t*-tests. A value of *p* < 0.05 was considered statistically significant.

## Results

### SA pretreatment lowers t-BHP-induced ROS production in HepG2 cells

**Figure 1.**
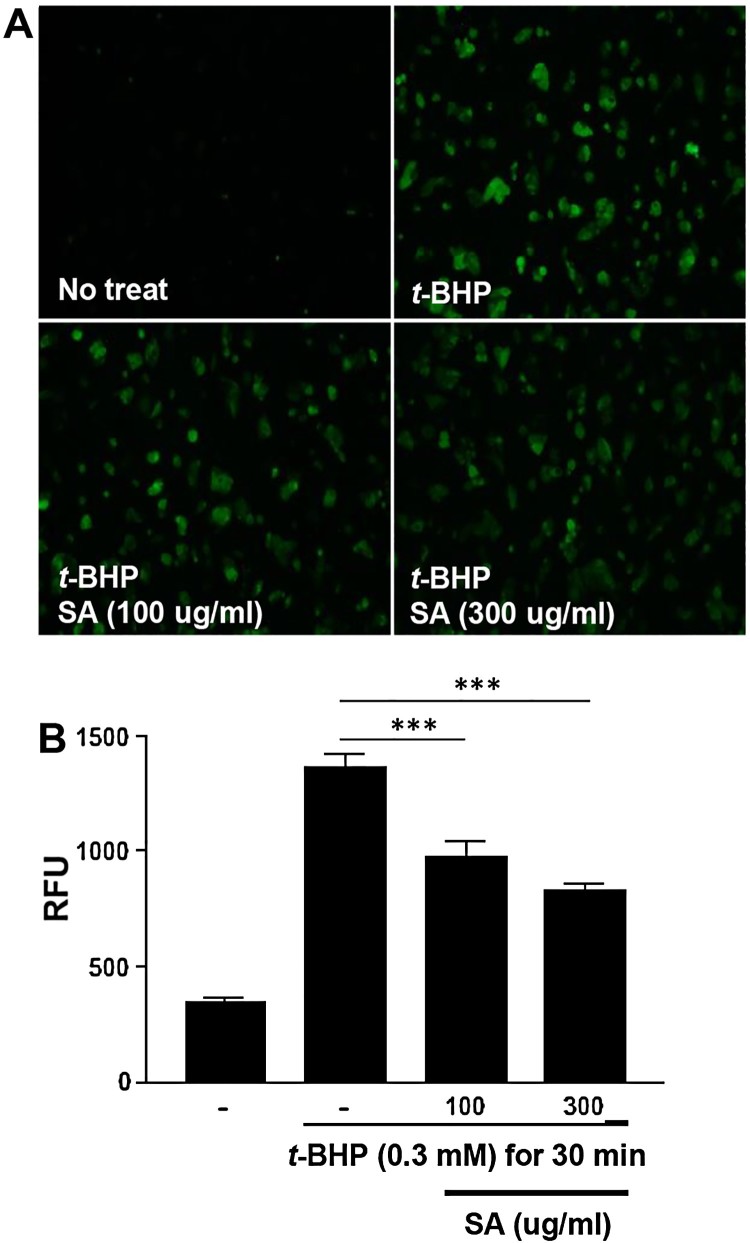
Effects of SA on ROS production in *t*-BHP-treated HepG2 cell. (A) The effects of SA on ROS production in *t*-BHP-treated HepG2 cells in 6-well plates were monitored using a florescence microscope (magnification 100×). (B) Total ROS production of *t*-BHP-treated HepG2 cells was measured using a fluorometer. The data are shown as the means ± SEM of three indicated experiments. *** *p* < 0.001 (Student’s *t*-test).

Total ROS production was measured in the HepG2 cells after 0.3 mM *t*-BHP treatment. An increase in ROS production was observed in HepG2 cells treated with 0.3 mM *t*-BHP more than in the untreated controls. However, the cells pretreated with SA showed a significantly lower ROS production than cells treated only with *t*-BHP (*p* < 0.05) (Figure 1(A and B)). These results suggested that SA pretreatment decreased *t*-BHP-induced ROS production in HepG2 cells.

### SA pretreatment blocks ROS production and lowers ALT levels in the Con A-induced liver injury model

**Figure 2.**
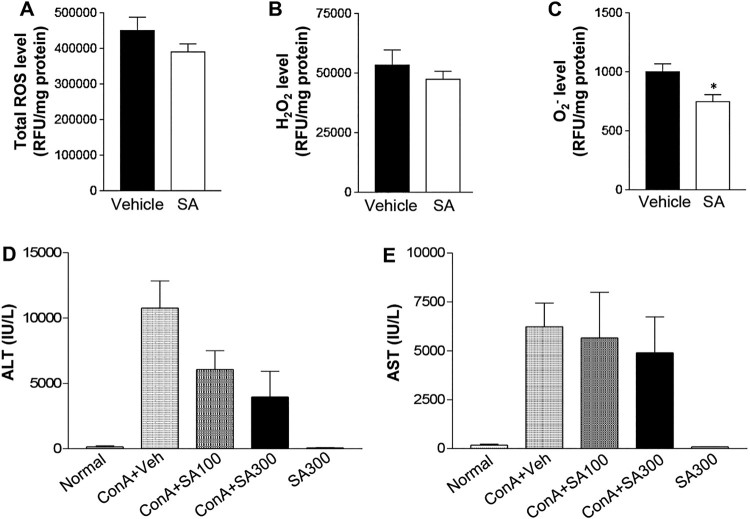
Effects of SA on ROS production in the liver after Con A exposure. Mice were subjected to Con A (15 mg/kg, IV) treatment with or without SA (IP). (A) Total production of reactive oxygen species (ROS) in the hepatic cytosolic fractions from vehicle- or SA-treated mice was assayed by measuring the fluorescence produced by 20 μM DCFH-DA. (B) H_2_O_2_ and (C) O^2-^ production were estimated in the hepatic cytosolic fractions of liver from vehicle (*n* = 5) and SA treated mice (*n* = 5). *p < 0.05 compared to Con A + Veh mice by Student’s *t*-test. (D) ALT and (E) AST levels were measured 6 h after Con A injection in mice. Normal: non-treated group (*n* = 5), Con A + Veh (*n* = 5): Vehicle- and Con A-treated group (*n* = 5), Con A + SA100 (*n* = 5): SA (100 mg/kg)- and Con A-treated group, Con A + SA300 (*n* = 5): SA (300 mg/kg)- and Con A-treated group.

To examine the effects of SA single dose on ROS production, mice were pretreated with 300 mg/kg SA [10] and hepatic total ROS, H_2_O_2_, and $O_{2}^{-}$ levels were measured at 6 h after Con A injection. In the livers of mice pretreated with SA, although the total ROS and H_2_O_2_ levels were not statistically different, both levels were lower than in the vehicle group (Figure 2(A and B)). Interestingly, the Con A-induced increased levels of $O_{2}^{-}$ was significantly decreased by SA pretreatment (Figure 2(C)). Next, we measured ALT and AST levels to determine the dose-dependent effects of SA on Con A-induced liver injury. Plasma ALT and AST levels were highly increased by Con A treatment (Figure 2(D and E)). However, the Con A-mediated increase in plasma ALT level was dramatically improved by SA administration in a dose-dependent manner (Figure 2(D)). These results suggested that the SA extract exerted a potent antioxidant activity *in vitro* and *in vivo* and attenuated liver injury in Con A-induced animal models.

### Effect of SA pretreatment on antioxidant enzyme activities in the liver after Con A treatment

**Figure 3.**
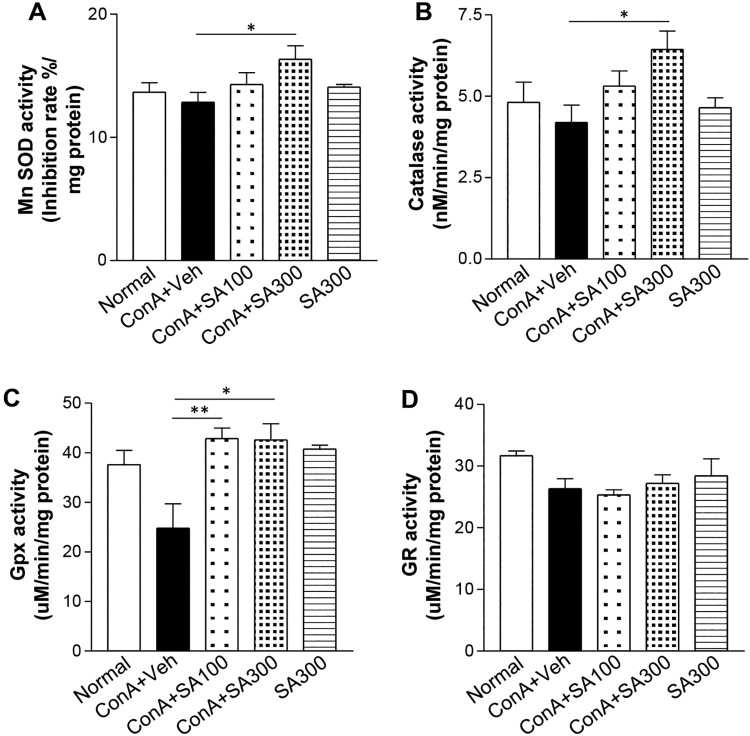
Effects of SA on intracellular antioxidant enzyme activities. Mice were treated with 15 mg/kg of Con A with or without SA (100, 300 mg/kg). Six hours after Con A exposure, liver samples were taken from the mice. (A) MnSOD, Mn superoxide dismutase; (B) CAT, catalase; (C) GPx, glutathione peroxidase; and (D) GR, glutathione reductase levels, were measured by enzymatic assays. * *p* < 0.05 or ** *p* < 0.01 compared with the group treated with Con A-Veh (Student’s *t*test).

Accumulated cellular ROS is eliminated by various antioxidant enzymes to maintain redox homeostasis [14]. Therefore, we assayed the activities of antioxidant enzymes in the liver tissues. MnSOD (Figure 3(A)), CAT (Figure 3(B)), and GPx (Figure 3(C)) activities were significantly increased in the Con A-SA group more than in the Con A group, but GR activities (Figure 3(D)) were not significantly different between the two groups. Moreover, SA-only treatment was not able to affect the activities of antioxidant enzymes. These results suggested that SA treatment ameliorated ROS by activating the antioxidant enzymes SOD, CAT, and GPx.

### SA pretreatment inhibits liver histological alterations after Con A treatment

**Figure 4.**
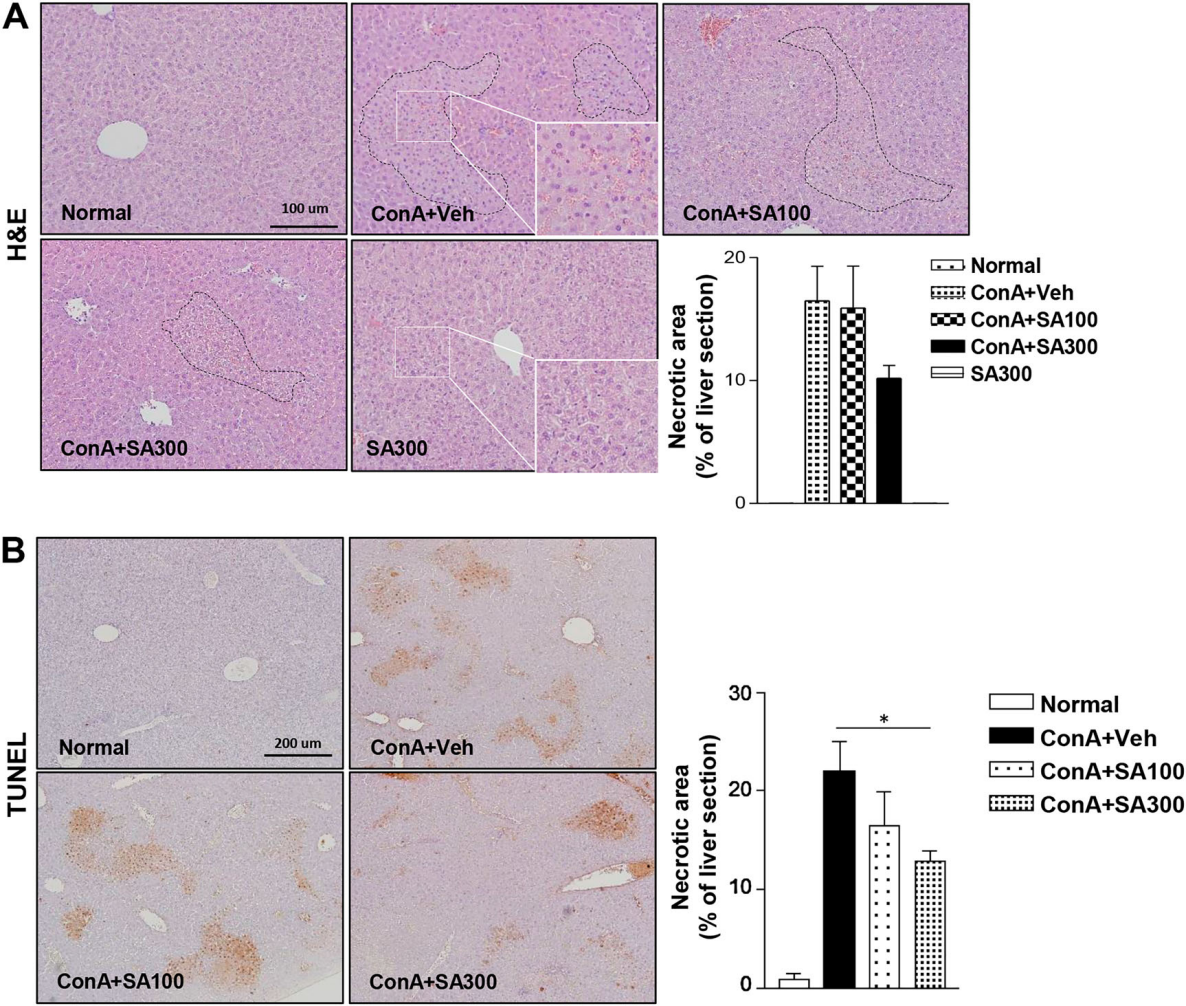
Effect of SA on histological alterations and apoptosis in the liver after Con A exposure. Histological assessments were performed by H&E (magnification: 200× or 400×) (A) and TUNEL staining to detect apoptotic cells (magnification: 100×) (B). Scale bars, 100 μm or 200 μm. *p < 0.05 compared to Con A + Veh mice by Student’s *t*-test. Injury area and necrotic area presented as the percentage of liver section.

To evaluate the protective effects of SA against Con A-induced liver injury, an *in vivo* experiment was performed. Histological abnormalities were detected in the livers of Con A-treated mice; however, the H&E-stained liver tissues of the SA-pretreated mice (Figure 4(A)) showed low cell death. The TUNEL assay (Figure 4(B)) showed that Con A-induced apoptosis was significantly ameliorated by SA pretreatment in a dose-dependent manner (*p* < 0.05). These findings are consistent with the plasma ALT and AST levels.

### SA pretreatment lowers cell apoptosis markers and neutrophil infiltration induced by Con A in HepG2 cells

**Figure 5.**
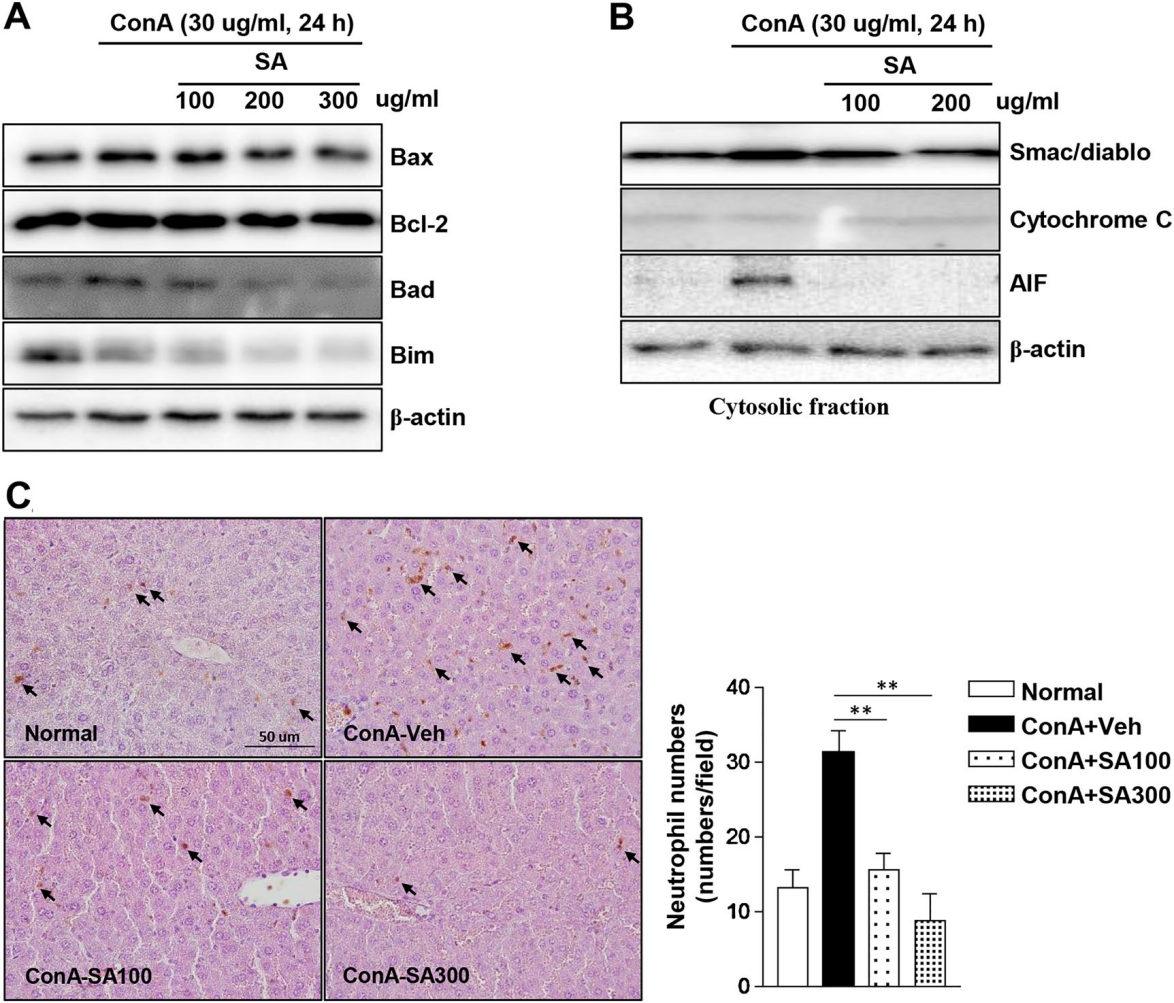
Effects of SA on apoptotic protein levels and neutrophil infiltration in Con A-treated HepG2 cells. The expression levels of apoptotic proteins was measured by western blotting. Concentration-dependent induction of (A) markers of apoptosis (Bax, Bcl-2, Bad, and Bim) in the HepG2 cells following treatment with SA and (B) mitochondrial release of apoptosis-related proteins (Smac/DIABLO, cytochrome C, and AIF) were detected in the cytosolic cell fractions by western blotting. β-actin was used as a loading control for each experiment. The data are representatives of three independent experiments. (C) Histological assessment was performed by immunohistochemical staining using an antibody against neutrophils, and representative images are displayed (original magnification: 100×). Arrows indicate neutrophils. Neutrophil counts in the liver tissues (right grape). ** Significantly different from the group treated with Con A-Veh, *p* < 0.01 (Student’s *t*-test).

To evaluate the effect of SA against Con A-induced cell apoptosis, HepG2 cells were pretreated with SA at the indicated concentrations and cell apoptosis was stimulated with Con A. Con A-induced increase in apoptotic markers (Bax, Bad, and Bim) levels were decreased by SA in a concentration-dependent manner. However, the level of Bcl-2, an apoptosis inhibitor, was not affected by SA (Figure 5(A)). Next, we confirmed the levels of necrotic markers because necrosis is a major mode of cell death in liver injury. Smac/DIABLO and AIF levels were dramatically induced by Con A treatment. However, this cytosolic release was completely blocked by SA pretreatment (Figure 5(B)). Subsequently, Con A-induced neutrophil accumulation was significantly reduced by SA extract pretreatment in a dose-dependent manner (Figure 5(C)). These findings suggested that SA inhibited both Con A-induced apoptosis and necrosis in HepG2 cells.

## Discussion

Hepatitis has many causes, such as viral infection, autoimmunity, alcohol, and drugs, and this disease is a threat to human health and is a worldwide problem [15]. The mortality rate of liver failure is as high as 50% [16] and therapeutic approaches to recover liver function are still limited. Therefore, finding novel drugs for the prevention and treatment of liver injury is of great interest. Many plant extracts have been examined for their ability to prevent liver injury [17–19]. In this study, we investigated the effect of SA as a potent candidate for the treatment of liver injury.

Oxidative stress is one of the prominent causes of liver injury mediated by various factors, including infection [20], autoimmunity [21], alcohol [22], free fatty acids [23], and drugs [24]. ROS may cause damage at the cellular level via membrane lipid peroxidation, cell degeneration, apoptosis, and necrosis [25]. In the current study, we investigated whether the ethanolic SA extract can suppress ROS overproduction. We found that the ethanolic SA extract decreased the *t*-BHP-induced increase in ROS production in cultured cells, which has often been used as a model for investigating the mechanism of cell injury initiated by acute oxidative stress [11]. Consistent with the *in vitro* data, our *in vivo* study also showed that in the SA-pretreated group, ROS levels were lower in the liver more than in the vehicle-treated and Con A-treated groups. Consistent with this, SA treatment significantly attenuated Con A-induced increases in serum ALT and AST levels, which are common markers of liver injury, indicating that SA exerted a potent antioxidant activity and suppressed acute liver injury.

The antioxidant system is essential for cellular responses to cope with oxidative stress, and antioxidant enzymes, such as CAT, SOD, and GPx, are used as indexes to evaluate the level of oxidative stress [22]. In our present study, SA pretreatment significantly increased the activities of SOD, CAT, and GPx, but not GR activity. These findings suggested that SA treatment reduced oxidative stress by activating antioxidant enzymes.

Con A is widely used in the investigation of immune-mediated acute liver injury [26]. Con A-induced hepatitis mimics many aspects of human acute liver failure, including severe damage by activated T and natural killer T (NKT) cells [27]. Con A-stimulated T cells produce TNF-α [28] and TNF-α induces apoptosis in mouse hepatocytes by stimulating ROS production [29]. We also observed increased apoptosis in the liver of mice treated with Con A. Interestingly, Con A-induced cell death was significantly improved by pretreatment with SA extract. Furthermore, Con A-induced increases in pro-apoptotic protein (Bax, Bad, and Bim) levels were dramatically decreased in SA-treated HepG2 cell lines in a concentration-dependent manner. Furthermore, the cytosolic release of Smac/DIABLO and AIF was decreased by pretreatment with SA. These findings suggested that SA extract inhibited Con A-induced cell death by suppressing ROS.

There were some limitations in this study. First, this study lacked any specific information of the active compounds in the SA extracts. Second, we were unable to evaluate the underlying mechanism how SA extracts regulate anti-oxidative enzymes and improve Con A-induced liver injury. Previous study on chemical constituents from SA reported that some flavonoids were contained in this plant [30]. Flavonoids are a large group of natural componds with phenol rings found in foods and beverages of plant origin. It has been reported that this compounds have an anti-oxidant activity and prevent human diseases [31]. However, in current states, we need to investigate the effect of active compounds for the anti-oxidant property of SA.

In summary, the current study highlighted the hepatoprotective effects of SA on Con A-induced acute liver injury by reducing liver injury markers, cell death, and cell death-related protein levels. This beneficial effect of SA was initially mediated through ROS inhibition by enhancing hepatic antioxidant enzyme activity, which led to the prevention of cell death and neutrophil infiltration. Although further research should be performed to identify and characterize the active compounds of SA extracts, the current findings have shown that SA extract administration might be a potential therapeutic strategy for patients with liver injury.
